# Global Proteomic Profile of Aluminum-Induced Hippocampal Impairments in Rats: Are Low Doses of Aluminum Really Safe?

**DOI:** 10.3390/ijms232012523

**Published:** 2022-10-19

**Authors:** Leonardo Oliveira Bittencourt, Rakhel Dayanne Damasceno-Silva, Walessa Alana Bragança Aragão, Luciana Eiró-Quirino, Ana Carolina Alves Oliveira, Rafael Monteiro Fernandes, Marco Aurelio M. Freire, Sabrina Carvalho Cartágenes, Aline Dionizio, Marília Afonso Rabelo Buzalaf, Juliana Silva Cassoli, Ana Cirovic, Aleksandar Cirovic, Cristiane do Socorro Ferraz Maia, Rafael Rodrigues Lima

**Affiliations:** 1 Laboratory of Functional and Structural Biology, Institute of Biological Sciences, Federal University of Pará, Belém 66075110, Brazil; 2 Graduate Program in Health and Society, Faculty of Health Sciences, State University of Rio Grande do Norte (UERN), Mossoro 59610210, Brazil; 3 Laboratory of Pharmacology of Inflammation and Behavior, Institute of Health Sciences, Federal University of Pará, Belém 66075110, Brazil; 4 Department of Biological Sciences, Bauru School of Dentistry, University of São Paulo, Bauru 05508060, Brazil; 5 Institute of Biological Sciences, Federal University of Pará, Belém 66075110, Brazil; 6 Faculty of Medicine, Institute of Anatomy, University of Belgrade, 11000 Belgrade, Serbia

**Keywords:** aluminum, hippocampus, neurotoxicity

## Abstract

Hippocampus is the brain area where aluminum (Al) accumulates in abundance and is widely associated with learning and memory. In the present study, we evaluate behavioral, tissue, and proteomic changes in the hippocampus of Wistar rats caused by exposure to doses that mimic human consumption of aluminum chloride (AlCl_3_) in urban areas. For this, male Wistar rats were divided into two groups: Control (distilled water) and AlCl_3_ (8.3 mg/kg/day), both groups were exposed orally for 60 days. After the Al exposure protocol, cognitive functions were assessed by the Water maze test, followed by a collection for analysis of the global proteomic profile of the hippocampus by mass spectrometry. Aside from proteomic analysis, we performed a histological analysis of the hippocampus, to the determination of cell body density by cresyl violet staining in *Cornu Ammonis* fields (CA) 1 and 3, and hilus regions. Our results indicated that exposure to low doses of aluminum chloride triggered a decreased cognitive performance in learning and memory, being associated with the deregulation of proteins expression, mainly those related to the regulation of the cytoskeleton, cellular metabolism, mitochondrial activity, redox regulation, nervous system regulation, and synaptic signaling, reduced cell body density in CA1, CA3, and hilus.

## 1. Introduction

The human environment is broadly polluted with aluminum (Al), the third most abundant element in the Earth’s crust, frequently found in combination with oxygen and other elements in bedrocks, soil, and sediments [1]. It is known for use in the production of household items, pans, packaging, construction, and transportation [2]; next, Al is used for vaccines manufacturing, where it performs the function of adjuvant to stabilize and preserve them [3] and also in the process of water treatment for removal of particulate materials [4]. Its exposure can be environmentally associated with acid rain, due to its high solubility capacity in acidic environments, and ultimately can lead to high levels of Al in plants, spices, cereals, vegetables, or grains, which can later be used for food products such as teas, food additives, coffee or granular products [5]. Other existing forms of Al exposure are mining, Al production, coal combustion, waste incineration, and motor vehicle exhaust, in addition to ingestion of water and cooking foods [6,7].

Al has no biological function in humans; moreover, prolonged exposure to Al may be associated with hematopoietic, skeletal, respiratory, immunological, neurological, and functional adverse effects [8]. Since Al-induced toxic effects are evidenced in vitro [9], in humans [10], and animal studies [11], this element has received considerable attention mainly due to its neurotoxic abilities. These effects are prominent and have been associated with neurodegenerative diseases, especially Alzheimer’s disease (AD) [12,13]. Studies suggest that the incidence of AD increased significantly in the elderly after prolonged ingestion of water with high concentrations of Al [14]. The possible molecular mechanisms involved in the Al-induced toxicity process are oxidative stress, cytotoxicity, genotoxicity, pro-inflammatory effects, enzyme dysfunctions, metabolic disturbances, membrane disruption, microtubule disruption, necrosis, and apoptosis [15,16,17].

As is the case for many brain areas, Al accumulates and compromises the hippocampus, a region mainly associated with learning and memory [18]. The hippocampus (also known as Ammon’s horn or *Cornu Ammonis,* (CA) occupies most of the floor in the inferior (temporal) horn of the lateral ventricle and is responsible for cognitive processes such as episodic memory formation and storage, spatial memory, working memory, and learning and memory storage [19]. The hippocampus is a trilaminate archicortex whose main histological feature is a single pyramidal cell layer. It could be divided into three distinct fields, known as CA1, CA2, and CA3. Other surrounding gray matters that are also included in the hippocampal formation are the subicular complex, dentate gyrus (DG), and hilum its hilum (CA4), belonging to the DG [20,21]. Studies showed that Al exposure impairs learning and memory capacity in both human and animal models [22,23,24].

In the present study, we aimed to analyze the effects of prolonged exposure to aluminum chloride (AlCl_3_) in doses that mimic the dosage allowed for human consumption in the hippocampus, as well as to investigate changes in the global proteome, tissue changes in neuronal cell body density in CA1, CA3 and hilum regions, and also cognitive changes. The hypothesis is that this exposure may be correlated to proteomic alterations and hippocampal neurodegeneration, resulting in learning and memory deficits.

## 2. Results

### 2.1. The Global Proteomic Profile of Rats’ Hippocampus Was Significantly Affected by Long-Term and Low-Dose Aluminum Exposure

A total of 215 proteins showed different regulation statuses in the hippocampus of rats. Among these proteins, 22 were uniquely identified in the control group and 72 in the exposed group. In addition, the expression of one protein was downregulated, and the expression of 214 was upregulated as fully described in Supplementary Materials (Tables S1 and S2).

**Figure 1:**
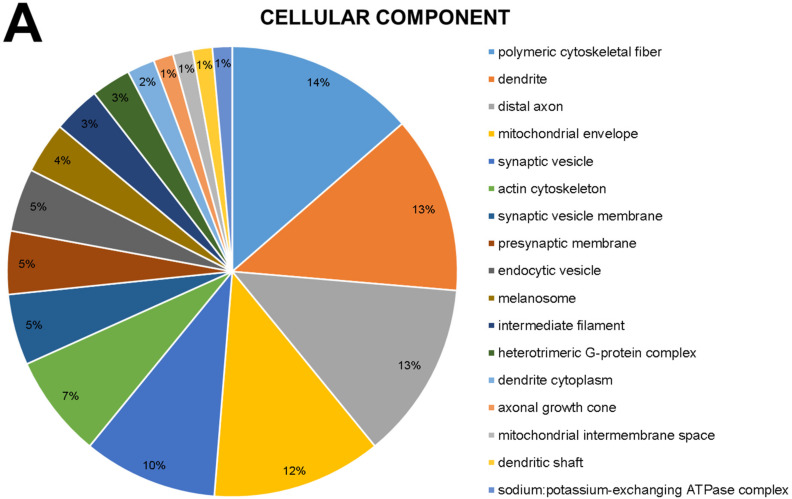

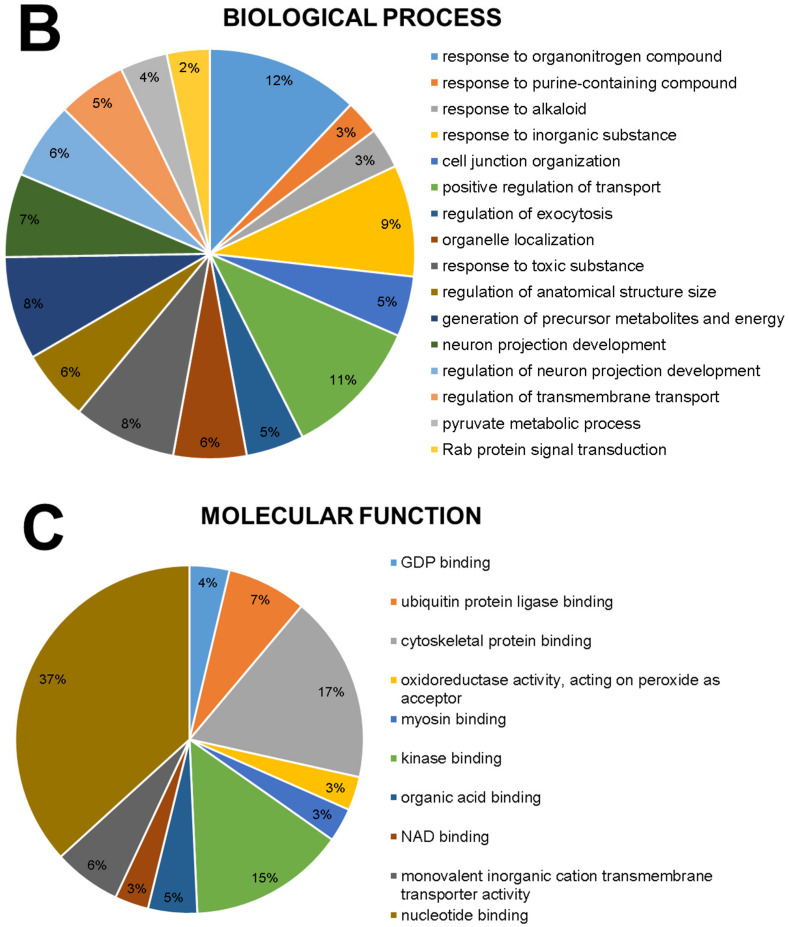
Distribution of proteins with different expressions in (**A**) Cellular Component (**B**) Biological Process (**C**) Molecular Function of rats exposed to AlCl_3_ vs. control. Categories of proteins are based on Gene Ontology annotation of the biological process. Protein accession numbers were provided by Uniprot.org. The gene ontology was evaluated according to the ClueGo plugin of Cytoscapes software 3.6.

According to Gene Ontology, the modulated proteins are related to 17 cellular components, where six are most affected: polymeric cytoskeletal fiber (14%), dendrite (13%), distal axon (13%), mitochondrial envelope (12%), synaptic vesicle (10%) and actin cytoskeleton (7%) (Figure 1A).

About biological processes, 16 processes are involved, where we can point out response to organonitrogen compound (12%), positive regulation of transport (11%), response to an inorganic substance (9%), generation of precursor metabolites and energy (8%) and response to a toxic substance (8%) (Figure 1B).

About molecular functions, 10 functions have been reported (Figure 1C): nucleotide binding (37%), cytoskeletal protein binding (17%), kinase binding (15%), ubiquitin protein ligase binding (7%), and monovalent inorganic cation transmembrane transporter activity (6%).

**Figure 2:**
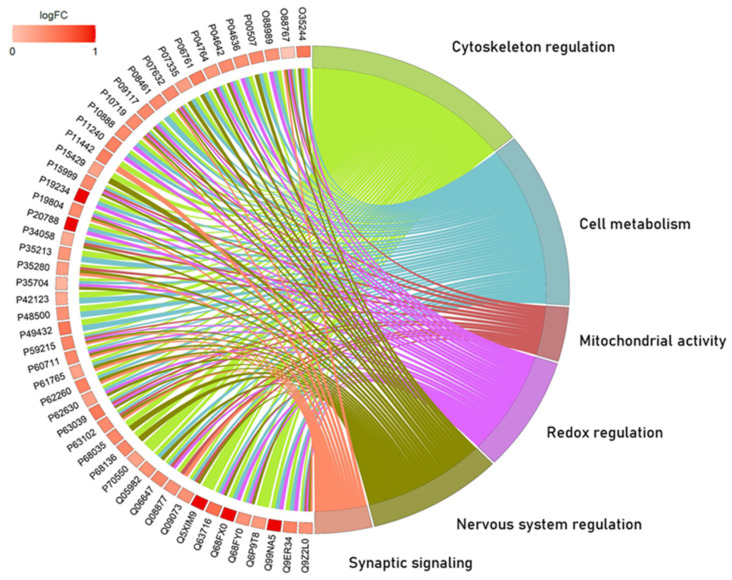
Over-representation analysis (Circos plot) presents the protein-protein interactions between the AlCl_3_ and control groups in the hippocampus and the biological processes involved according to Gene Ontology. The altered regulation is expressed according to the colors. Red represents superexpression, and light red represents subexpression.

Over-representation analysis showed the interactions of 50 proteins (fully described in Supplementary Table S3) grouped into six major biological processes according to Gene Ontology: cytoskeleton regulation, cell metabolism, mitochondrial activity, redox regulation, nervous system regulation, and synaptic signaling (Figure 2).

### 2.2. Aluminum-Exposed Rats Displayed Morphological Damages in the Hippocampus

**Figure 3:**
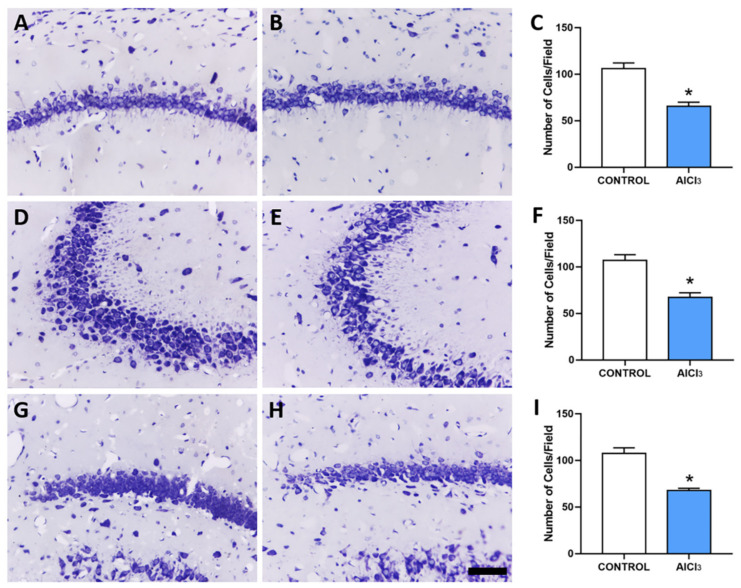
Aluminum exposure is associated with the decreased density of the hippocampal cell body of rats. Cresyl violet cell body density count in CA1 (**A**): control, (**B**): AlCl_3_, and (**C**): cell counting), CA3 (**D**): control, (**E**): AlCl_3_, and (**F**): cell counting), and hilus hippocampal regions (**G**): control, (**H**): AlCl_3_, and (**I**): cell counting). * *p* < 0.05, Student’s *t*-test, 12 animals per group. Scale bar = 50 µm.

The exposure to AlCl_3_ (8.3 mg/kg/day) over 60 days induced cell death in hippocampal regions when compared with the control group. Thus, in the Figure 3, we can see that Al reduced significantly the density of hippocampal cell bodies in CA1 (control: 106.2 ± 2.455 cells/field; AlCl_3_: 65.5 ± 1.875 cells/field; *p* < 0.0001), CA3 (control: 107 ± 2.582 cells/field; AlCl_3_: 67.33 ± 2.011 cells/field; *p* < 0.0001), and hilus (control: 107.7 ± 2.445 cells/field; AlCl_3_: 67.83 ± 0.9458; *p* < 0.0001).

### 2.3. This Prolonged Exposure to Aluminum at Doses Equivalent to Urban Regions Was Associated with Cognitive Impairment in Rats

**Figure 4:**
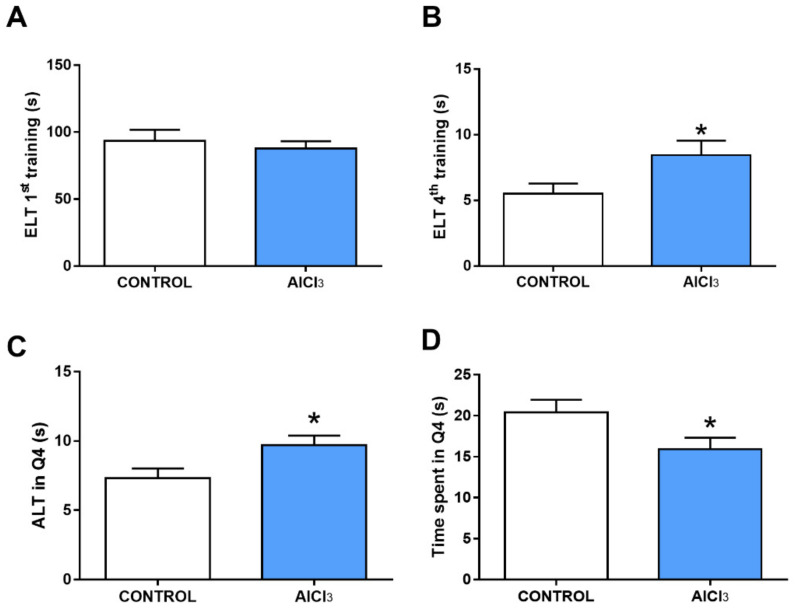
Aluminum exposure is associated with increased learning deficiency and spatial memory in rats. In (**A**) and (**B**) Escape latency time (ELT) first and fourth training in seconds. (**C**) Latency to arrive (ALT) in the target quadrant (Q4) and (**D**) time spent in target (s), respectively. Results are expressed as mean ± standard error of the mean. * *p* < 0.05, Student’s *t*-test, 12 animals per group.

Our data demonstrate that Al exposure increased the learning and memory deficit of the rats (Figure 4). In the first training (Figure 4A), there was no significant difference between the groups (control: 93.4 ± 8.3 s; AlCl_3_: 87.78 ± 5.415 s; *p* = 0.5872); however, in the fourth training (Figure 4B), the animals of the Al group showed a lower performance when compared with the control group (control: 5.5 ± 0.7782 s; AlCl_3_: 8.444 ± 1.107 s; *p* = 0.04), requiring more time to reach the target quadrant (Q4). Regarding memory, after 24 h of testing, the animals in the Al group increased their latency time of arrival at the target quadrant (ALT) as compared to the control group (control: 7.3 ± 0.7157 s; AlCl_3_: 9.7 ± 0.7 s; *p* = 0.0276; Figure 4C). Regarding the time spent in Q4, the Al group decreased this time when compared with the control group (control: 20.4 ± 1.558 s; AlCl_3_: 15.9 ± 1.418 s; *p* = 0.0466; Figure 4D).

## 3. Discussion

To the best of our knowledge, this is the first study bringing the proteomic approach underlying the urban-like Al exposure levels in aluminum-induced cognitive impairments in a rat experimental model. Comprising the interface between molecular changes observed by proteomic, and behavioral changes, the morphological assessment also revealed significant changes in CA1, CA3, and hilus regions in the dorsal hippocampus, being suggestive of a neurodegenerative process. The following findings that will be discussed, bring new and important evidence of Al neurotoxicity and open new horizons towards the association between Al and cognitive-related diseases.

Due to its constant presence in the environment, humans can be directly and indirectly exposed to Al daily, whether in cosmetics, constructions, dental products, packaging articles, medicines, food, and water, through skin contact, inhalation, or oral ingestion [25]. In this perspective, considering the multiple sources of Al that humans are exposed to, the World Health Organization (WHO) and European Food Safety Authority have delimited the tolerable weekly intake (TWI) of 2 mg/kg and 1 mg/kg, respectively, and the No Observed Adverse Effect Level (NOAEL) of 30 mg/kg/day [26,27]. Then, using the allometric approach to perform an experimental study with higher translational meaning as previously described [28], we obtain the values of 6.25–12.5 mg/kg/week of an Al intake, and the NOAEL of 187.5 mg/kg/day for studies in rats. The dose of 8.3 mg/kg/day used in our study is equivalent to 1.67 mg/kg/day of Al, which results in a lower weekly intake than the TWI determined by WHO, and is around 100 times lower than the NOAEL. This highlight not only the translational meaning of this study but reinforce that such values must be treated as ‘provisional’ and that need to be reviewed constantly.

In a previous study from our group, this dose of 8.3 mg/kg/day was able of causing neurological alterations in rats [16]. However, it is worth mentioning that such findings were not associated with higher Al content in hippocampal parenchyma in comparison to control, suggesting that, although higher levels of Al were not found in the hippocampus, the neurological damage may have resulted from secondary biological events triggered by the increase in blood bioavailability shown in [29], triggered by the Al presence in the bloodstream and its crossing through the blood-brain barrier by binding to the plasma protein transferrin [30].

Mechanisms of Al-induced neurotoxicity as the basis for the appearance of related neurodegenerative conditions are already described in the literature [31]. Thus, considering the possible association of Al with cellular involvement, we evaluated cell density and found that there was a decrease in the CA1, CA3 and hilus regions. Similar results reveal in evaluation studies in the same regions of the hippocampus after exposure to other metals and environmental pollutants, as well as cognitive damage, since the areas of CA1, CA3, and hilus of the hippocampus are related to learning and memory processes [19,32]. On the other hand, we hypothesize that side decreased density of hippocampal cell bodies could lead to gradual hippocampal atrophy, whereas hippocampal atrophy is a substrate of various diseases such as depression and AD [33,34]. Studies suggest that Al promotes neurotoxicity [35], causing cognitive damage such as memory and learning through neuronal loss [36,37].

The behavioral effects described in this work may be related to Al-induced damage in areas of the central nervous system (CNS) that are important for learning and memory. In the other work of our group, this possibility was assessed from neuronal density and glial fibrillary acidic protein (GFAP) immunoreactivity in the CA1 and CA3 regions of the hippocampus [37]. A decrease in CA1 and CA3 neuron density and a progressive decrease in GFAP immunoreactivity were seen in animals intoxicated with Al. These results are supported by previous in vitro and in vivo studies showing damage in the hippocampus after Al intoxication. These neurotoxic effects of Al can be justified by the tropism that this metal has for CNS structures [38,39]. There is evidence in the literature that points to several damages to the CNS due to Al intoxication, causing oxidative damage in cells of the hippocampus.

Cognitive functions involve various regions of the nervous system with particular characteristics and inherent biochemical processes. Among these functions are those associated with memory and learning, which in turn are managed by the hippocampus [40]. In turn, the hippocampus is a region capable of consolidating the processes of storage and retrieval of events [41]. Previous evidence has shown that Al exposure promotes deficits in aversive memory and changes in the ability to recognize objects [16]. In our study, memory and learning were analyzed using the water maze test, which is one of the most used analysis tools in behavioral neuroscience, allowing the assessment of long and short-term spatial learning and memory [42]. Learning is assessed through repeated trials and spatial memory is assessed by a preference for the platform area when the platform is absent [43].

The water maze test, which presupposes the spatial orientation of survival inside the pool and escapes from the water, has been widely used as a behavioral test for the evaluation of memory and learning, which are based on the synaptic plasticity of the hippocampus, involved in spatial memory. It is understood, therefore, that the performance of animals in the task is critically dependent on the integrity of the hippocampus [44]. A study of exposure to lead (Pb) in rats promotes deficits in the performance of this task. Corroborating this result, the same study shows a reduction in the density of dendritic spines of CA1 and impairment of LTP in CA3 and CA1, reinforcing the correlation between the behavioral results obtained and the importance of the areas studied [45]. The findings of this behavioral test showed that exposure to Al promoted a learning deficit in animals with a longer action time to reach the target quadrant in the test. Likewise, the animals exposed to Al showed a cognitive impairment related to long-term memory in which we observed that the animals had a longer latency time of arrival in the target quadrant.

When we discuss the hippocampal cognitive functions and the role of different neuron populations in the structure, we immediately need to consider the CA1, CA3, and hilus regions analyzed in our study. The CA1 and CA3 present pyramidal neurons called “place cells”, which refer to the mnemonic and navigation processes attributed to the hippocampus [46]. During the task of learning and recognizing a new environment with the spatial memory paradigm, the CA3 is associated with the first stage of memory encoding, while the CA1 is linked with memory consolidation [47], receiving afferent signs from CA3 through the collateral Shaffer pathway [48]. In addition, it is important to take into account the role of neuroplasticity in cognitive processes, which can be illustrated by synaptic plasticity and neurogenesis present in the hippocampus. The third region analyzed inhere was the hilus, which presents a large number of mossy cells, but also GABAergic neurons, playing important roles in the commissural/associational pathway [49]. Indeed, we are aware that the cresyl violet technic presents several limitations, however, our data is in accordance with the Al literature regarding hippocampal neurodegeneration [50,51], then our data suggest that low doses of Al may cause neural cell death.

Beyond the increase in Al bioavailability, it is important to take into account the background of molecular and biochemical features underlying Al-induced neurodegeneration. For that, we performed the global proteomic profile screening, to identify proteins that would indicate possible targets of Al neurotoxicity. The proteomic approach by mass spectrometry is one of the tools commonly used to describe proteins with a differential pattern of expression and has been used in toxicology sciences to describe potential biomarkers or predictors of damage [52,53,54]. The Gene Ontology analyses of the Al-exposed hippocampal proteome revealed the modulation of important components in neural physiology. In cellular components, Figure 1A showed components related to energy metabolism, synaptic communication, and axonal morphology, e.g., The biological processes (Figure 1B), on the other hand, demonstrated hippocampal response to stimuli, and the molecular function (Figure 1C) showed functions associated to vesicle transporting and proteostasis, for example. Such diversity of categories led us to perform the over-representation (ORA) analysis, which allowed us to identify the exact protein (or proteins) involved in those processes.

According to Gene Ontology, modulated proteins are related to 17 cellular components, where six are most affected: polymeric cytoskeletal fiber (14%) which is nothing more than a cytoskeletal component consisting of a homo- or heteropolymeric fiber constructed from an indeterminate number of protein subunits. In addition, we have the term dendrite (13%) which is a neuronal projection that has a short, tapered, tapering morphology. Dendrites receive and integrate signals from other neurons or sensory stimuli, and conduct nerve impulses toward the axon or cell body. In most neurons, the impulse is transmitted from the dendrites to the axon via the cell body, but in some types of unipolar neurons, the impulse does not travel through the cell body. Also in cellular components, we found alterations of the distal axon gene (13%) which is the long process in a neuron that conducts nerve impulses, usually away from the cell body to the terminals and varicosities, which are sites of neurotransmitter storage and release; mitochondrial envelope (12%) that is the double lipid bilayer enclosing the mitochondrion and separating its contents from the cell cytoplasm, which includes the intermembrane space; the synaptic vesicle (10%) of presynaptic nerve terminals, that accumulates high concentrations of neurotransmitters and secretes these into the synaptic cleft by fusion with the active zone of the presynaptic plasma membrane and finally, actin cytoskeleton (7%) which is nothing more than a part of the cytoskeleton (the internal framework of a cell) composed of actin and associated proteins. Includes actin cytoskeleton-associated complexes (Figure 1A).

About biological processes, 16 processes are involved, in which we can point out the response to organonitrogen compound (12%), which is any process that results in a change in the state or activity of a cell or an organism (in terms of movement, secretion, enzyme production, gene expression, etc.) because of an organonitrogen stimulus. An organonitrogen compound is formally a compound containing at least one carbon-nitrogen bond. In addition, we find positive regulation of transport (11%) which is any process that activates or increases the frequency, rate, or extent of the directed movement of substances (such as macromolecules, small molecules, ions) into, out of, or within a cell, or between cells, of using some agent such as a transporter or pore. Still, in biological processes, we find a response to an inorganic substance (9%) which is any process that results in a change in the state or activity of a cell (in terms of movement, secretion, enzyme production, gene expression, etc.) as a result of an inorganic substance stimulus; generation of precursor metabolites and energy (8%), which is any process that modulates the frequency, rate or extent of the chemical reactions and pathways resulting in the formation of precursor metabolites, substances from which energy is derived, and the processes involved in the liberation of energy from these substances, and finally, a response to a toxic substance which deals with Any process that results in a change in state or activity of a cell (in terms of movement, secretion, enzyme production, gene expression, etc.) as a result of a toxic stimulus (8%) (Figure 1B).

About molecular functions, 10 functions have been reported that are about bindings (Figure 1C): nucleotide binding (37%) which is a binding to a purine nucleotide, a compound consisting of a purine nucleoside esterified with (ortho)phosphate, the cytoskeletal protein binding which is binding to ankyrin, a 200 kDa cytoskeletal protein that attaches other cytoskeletal proteins to integral membrane proteins (17%), the kinase binding which is binding to a kinase, any enzyme that catalyzes the transfer of a phosphate group (15%), ubiquitin protein ligase binding which is binding to a ubiquitin-protein ligase enzyme, any of the E3 proteins (7%), and finally the monovalent inorganic cation transmembrane transporter activity which is ‘di-, tri-valent inorganic cation transmembrane transporter activity’|‘divalent inorganic cation transmembrane transporter activity’|‘monovalent inorganic cation transmembrane transporter activity’|‘trivalent inorganic cation transmembrane transporter activity (6%).

The ORA pointed out six biological processes and the proteins related to them. In Figure 2 it is possible to note that most of the processes had more than two common proteins, which illustrate how cell physiology works by having an orchestrated signaling system. In the following paragraphs, we will address our discussion based on those six biological processes and highlight some proteins that may be underlying the behavioral and morphological findings previously discussed.

A factor that must be taken into account in proteomics studies is the action of anesthesia on protein expression. Both in vitro and in vivo studies demonstrate that prolonged action of anesthetics (i.e., ketamine, propofol, dexmedetomidine) modifies proteomics, ultimately leading to differential protein expression [55,56,57,58,59], with different types of anesthesia resulting in differential effects in distinct regions of the brain, including the hippocampus [59,60,61,62]. The use of anesthesia aimed at euthanasia has a varied effect depending on the anesthetic used and the protein analyzed, for instance, not resulting in a significant impact on MAP kinase protein expression [60]. However, since serum ketamine levels are highest at 10–20 min post-administration [63], we hypothesized that its use for immediate perfusion after its administration did not impact protein expression levels in the brain in our study. Nevertheless, studies comparing anesthetized and non-anesthetized animals are further required for a better understanding of the impact of anesthetic action in our experimental model.

As stated above, we believe that there is no interference in the proteomic analysis of anesthesia. First, because the animal in question does not die due to the amount of anesthetic applied, but rather due to cardiac perforation. Second, immediately after the animal is simply anesthetized and the animal’s reflexes disappear, the animal is euthanized, as already mentioned in the methodology session, by cardiac puncture and then the collection of these materials is performed. Third, considering the comparability between groups, all animals are subjected to the same procedures. Fourth, if another method of euthanasia were considered, it would also affect the molecular characteristics of the animals’ brains, such as the CO_2_ chamber. As for the number of publications describing the effects of anesthetics on the hippocampus, there are some that aim to mimic the long-term use of anesthetics, and their recreational and illegal use (see [64,65]), an approach beyond the scope of our study.

Finally, the doses of ketamine and xylazine used in this experiment were according to the weight of each animal and obeying the laws of the committees in force that govern good experimental practices. We also reiterate that the same anesthetic and the same dose patterns were used for both the exposed group and the control group, thus eliminating any type of possible bias.

In the last years, we have shown that xenobiotics such as mercury, lead, and fluoride, have an interesting ability to modulate the proteostasis system [52,53,66,67]. One complex involved in proteostasis is the ubiquitin-proteasome, which regulates the degradation of proteins, along with the Heat Shock Proteins (HSP), which also work on the degradation process, but have special functions in preventing the misfolding of proteins that could lead to the formation of protein aggregates [68,69]. Our proteomic approach showed the up-regulation of HSP-70 kDa (P0DMW0, P0DMW1, and P14659) and HSP-90 KDa subunits (P34058 and P82995). The role of preventing misfolding and protein aggregation gave special attention to HSP-70 and 90 due to their involvement in Alzheimer’s disease progression [70].

The up-regulation of HSP is often related to cell aging and the triggering of oxidative stress, one of the main mechanisms of damage to Al. Previously this experimental design was capable of increasing lipid peroxidation and reducing the antioxidant competence against peroxyl radicals and reducing catalase activity [16]. This mechanism has been exhaustively described in the literature, especially considering the influence on the Fenton reaction and its repercussions on reactive oxygen species [71]. Despite that, the low dose used in our study was associated with the modulation of several proteins involved in redox balance, as seen in ORA analysis, such as Superoxide Dismutase [Cu–Zn] up-regulation (P07632) and exclusive regulation of Peroxiredoxin-4 (Q9Z0V5) in the aluminum-intoxicated group, for example. We also found upregulation of IDH3A-Isocitrate dehydrogenase [NAD](Q99NA5) and UCRI-Cytochrome b-c1 complex subunit Rieske_ mitochondrial (P20788) proteins, both involved in mitochondrial energy metabolism. Proteomic studies, including one using copper in the hippocampus, have observed changes in mitochondrial metabolic proteins such as IDH3A (Q99NA5) associated with mitochondrial encephalopathy [72] and UCRI (P20788), suggesting that altered mitochondrial energy metabolism is possibly associated with increased damage [73]. In vitro studies observed that Al promotes damage to the mitochondrial membrane, suggesting that Al is involved in cellular neurotoxicity mechanisms [74].

Oxidative stress is a remarkable feature of neurodegenerative processes since it can cause synaptic communication impairment and even cell death [75]. Thus, based on our proteomic findings, we suggest that low concentrations of Al are capable of unbalancing the synaptic signaling by the up-regulation of Synaptotagmin-2 (P29101), Synapsin-2 (Q63537), and Synaptophysin (P07825) for example. Such findings may be underlying the behavioral damages found here, especially considering the role of glutamatergic and cholinergic systems in hippocampal cognitive functions and Alzheimer’s disease [76].

Additionally, the neural microenvironment also responds to xenobiotic injuries and neurodegenerative diseases by adopting an inflammatory profile, which can be visualized in the proteomic analysis by the absence of Prostaglandin reductase 2 (Q5BK81) in the exposed group. This protein is involved in the arachidonic acid metabolism pathway, and its down-regulation can be associated with a reduction of anti-inflammatory capacity [77].

Furthermore, we also found the up-regulation of α- and β-synuclein (P37377 and Q63754, respectively) that have been suggested as components of Lewis bodies and cerebrospinal fluid in Alzheimer’s and Parkinson’s diseases [78,79]. Besides that, another remarkable feature of such diseases is cell death, and in our proteomic approach, we found the exclusive regulation of Caspase-8 (Q9JHX4) in the Al-intoxicated group. This protein has already been associated with Al-induced neuron apoptosis by Fas/FasL and Caspase-3 activation [80].

Thus, the proteomic analysis successfully showed molecular features underlying the morphological and behavioral damage visualized in the experimental assessments, which led us to hypothesize that even low levels of Al exposure may pose a threat to human health. These data must be interpreted with caution, but certainly, open new experimental questions about the association between human daily life Al exposure and neurodegenerative diseases.

## 4. Materials and Methods

### 4.1. Ethical Aspects and Experimental Design

Twenty-four male rats (*Rattus norvegicus*, Wistar strain), weighing between 120 g and 150 g, 90 days old, obtained from the Federal University of Pará Animal Facility (CEUA-UFPA; protocol number 5923210617) were used in the present study. The animals were kept in polypropylene collective cages (4 animals per cage), receiving water and food ad libitum.

The animals were divided into the exposed group (12 animals), which received a daily dose of 8.3 mg/kg of aluminum chloride (AlCl_3_; Sigma-Aldrich, St. Louis, MI, USA) solubilized in distilled water and administered by intragastric gavage for 60 days; and the control group (12 animals), which received only the vehicle (H_2_O_d_) by the same route and period of exposure.

The dose used in the present study was established and described in previous studies by our group, corresponding to 1.5 mg/kg/day of the accepted dose for the human diet [16,29,81,82]. Allometric calculation conversion was necessary, considering the biological differences between humans and rodents [82,83].

### 4.2. Water Maze Test

Twenty-four hours after the exposure period, the animals were selected randomly and taken to the behavioral testing room with attenuated sound and controlled lighting and temperature. The animals were habituated to the testing room for a minimum of 1 h before the beginning of the water maze test.

This test was used to assess learning and spatial memory, following a previously published protocol [32,84] adapted from Morris [85]. The task comprised a circular pool filled with water (25 °C) up to 30 cm, divided into four equal quadrants (Q1–Q4). A 29 cm high platform (10 cm^2^) made of transparent acrylic was placed in the center of the target quadrant (Q4) for the training session and removed from the pool 24 h after training. Each animal was subjected to four consecutive training sessions with 5 min between each session; after each session, the animal remained on the platform for 20 s. If the animal could not find the hidden platform within 120 s, it was gently led to the platform, where it stayed for 20 s. The time spent in each quadrant and the time needed to find the platform was recorded. On the test day (day 2), the animals were placed in the pool, without the platform, and allowed to explore the area for 60 s. The time needed to reach Q4, the quadrant in which the platform was on the training day, and the time spent in Q4 were recorded and analyzed.

### 4.3. Proteomic Analysis

After the behavioral assessment, a set of animals (n = 6/group) was deeply anesthetized with a solution of xylazine hydrochloride (10 mg/kg) and ketamine hydrochloride (100 mg/kg) (i.p.). Then, after the total abolishment of paw and corneal reflexes, the animals were euthanized by exsanguination. The hippocampus was collected after brain removal and gently washed in a cold saline solution. After, they were stored in microtubes, frozen in liquid nitrogen, and kept at −80 °C until further procedures.

The proteomic approach was carried out in biological triplicate by pooling two animals from the same group into one sample. Regarding pooling two samples into one, it is reasonable to state that the animals are from an isogenic strain, with the same age and sex. In addition, although pooling two animals into one single sample, we still performed a biological triplicate and further technical triplicate. After, the samples were submitted to a cryogenic mill, followed by protein extraction by buffer lysis under constant stirring and 4 °C. Then, a standardized protein concentration was determined by Bradford’s method (1 μg/μL), and the fixed volume of 50 μL was used for the following steps: alkylation, digestion, desalting, and elution of the peptides. The peptides’ reading and identification were performed using the nanoAcquity UPLC-Xevo Qtof MS system (Waters Co., Ltd. Milford, MA, USA). The proteomic data analyses were only possible due to the comparability between the samples within the same group, and the use of Protein Lynx GlobalSERVER software, robust software for data analyses, was able to determine the *p* values, hence the quantitative proteomic analysis, considering *p* < 0.05 for downregulated proteins and 1 − *p* > 0.95 for upregulated proteins. Detailed methodology is available elsewhere [11,66,86].

### 4.4. Bioinformatic Analysis

For the analyses, the Cytoscape software (version 3.8) [87] was downloaded and the spreadsheet containing all the protein’s accession IDs, description, fold changes, and respective *p* values were organized. The bioinformatics analyses were performed to identify the protein functions based on the Gene Ontology (GO) biological processes, using the ClueGO plugin of the Cytoscape software. Overrepresentation analysis (ORA) was performed as previously described [88], which used the EGSEA plugin of the R Studio software and Cytoscape with Enrichment Pipeline plugin. The circosplot with the biological processes was generated by the Goplot plugin in the R studio.

### 4.5. Morphological Analysis

After the behavioral test, the remaining set of animals (n = 6/group) was anesthetized with ketamine hydrochloride (90 mg/kg) and xylazine hydrochloride (10 mg/kg) and then perfused with 0.9% saline solution, heparinized, followed by 4% paraformaldehyde in 0.2 M phosphate buffer saline (PBS) [89]. The brains were post-fixed in Bouin’s solution for 4 h, processed and embedded in Paraplast^®^ (Sigma-Aldrich, St. Louis, MI, USA), and then cut at 5 µm with a microtome. We selected the dorsal hippocampus of the region of interest and elected the CA1, CA3, and Hilus areas as previously described [32,90].

**Figure 5:**
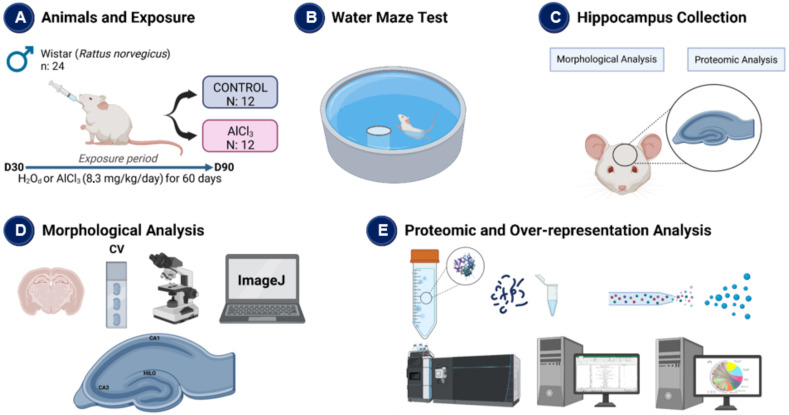
Methodological steps of the study. (**A**) Description of experimental groups and exposure to aluminum chloride (AlCl_3_) or distilled water (H_2_O_d_); (**B**) After the Al exposure protocol, the animals were submitted to the Water Maze Test; (**C**) After the Water maze test, the animals were euthanized, and the hippocampus was collected (**D**) Histological analysis with cresyl violet (CV) staining in the hippocampus, along the regions CA1, CA3 and hilus; (**E**) Proteomic analysis by mass spectrometry and over-representation.

After sample sectioning, we performed a histochemical analysis of cresyl violet (CV). While the former staining is used to stain Nissl substance in neurons, which are the rough endoplasmic reticulum, and it is a reliable method to estimate the neuron number according to the cell’s morphology. Next, the slides were mounted in gelatinized slides, dehydrated, and mounted on a coverslip with Entellan^®^ (Merck, Darmstadt, Germany). Photomicrographs were taken from three fields per region; each animal provided three different sections. Digital images were captured using a colored video camera attached to an optic microscope (Leica Microscope DM750, Leica Microsystems^®^, Heerbrugg, Switzerland) at magnifications of ×40 and analyzed using ImageJ software with a multi-point tool for cell counting (NIMH, NIH, Bethesda, MD, USA, https://imagej.nih.gov/ij/ accessed on 1 June 2022) [91,92,93]. All methodological procedures are summarized in Figure 5. 

### 4.6. Statistical Analysis

The results were tabulated and analyzed by GraphPad Prism 7.0 (GraphPad Software Inc., San Diego, CA, USA), using the Student’s *t*-test for behavioral and morphological analyses, with a significance level lower than 0.05. The results were expressed as mean ± standard error of the mean. The proteomic data were analyzed by the own software of the MS system, the Protein Lynx GlobalSERVER, after applying the Monte-Carlo algorithm.

## 5. Conclusions

This unprecedented study showed that even lower concentrations of aluminum in comparison to those doses considered tolerable can trigger significant molecular, morphological, and functional damages in rats’ hippocampus. The exploratory proteomic analysis showed the change in expression of proteins important for proteostasis, oxidative stress, cellular communication pathways, and cell death mechanisms, which resemble degenerative diseases. Although this is a preclinical study, it has a great translational appeal, and such findings highlight the need for attention to the safe limits determined by official regulatory agencies.

## Data Availability

All data are available within the article and in Supplementary Materials.

## Supplementary Materials

The following supporting information can be downloaded at: https://www.mdpi.com/article/10.3390/ijms232012523/s1.
