# Supplementary material for: Global Proteomic Profile of Aluminum-Induced Hippocampal Impairments in Rats: Are Low Doses of Aluminum Really Safe?
Source: Int J Mol Sci. 2022 Oct 19;23(20):12523. doi: 10.3390/ijms232012523 (PMC9603961; doi:10.3390/ijms232012523)
Supplement: Supplementary file 1 [file ijms-23-12523-s001.zip › ijms-1885084-supplementary.pdf]

International Journal of *Molecular Sciences*

MDPI

Supplementary Materials

Table S1. Identified proteins with expression significantly altered in the hippocampus of rats exposed to $AlCl_3$ vs. Control group.

Access Number[a]	Protein Name	PLGS Score	Fold Change	Biological Process
**P09811**	Glycogen phosphorylase_ liver form	16.73	1.68	CM
**P38406**	Guanine nucleotide-binding protein G(olf) subunit alpha	984.68	1.52	CM
**P04897**	Guanine nucleotide-binding protein G(i) subunit alpha-2	979.59	1.52	CM
**Q63803**	Guanine nucleotide-binding protein G(s) subunit alpha isoforms Xlas	979.59	1.52	CM
**P10824**	Guanine nucleotide-binding protein G(i) subunit alpha-1	979.59	1.51	CM
**P08753**	Guanine nucleotide-binding protein G(k) subunit alpha	979.59	1.51	CM
**P63095**	Guanine nucleotide-binding protein G(s) subunit alpha isoforms short	979.59	1.51	CM
**P29348**	Guanine nucleotide-binding protein G(t) subunit alpha-3	979.59	1.51	CM
**Q00729**	Histone H2B type 1-A	885.74	1.31	CM
**P04797**	Glyceraldehyde-3-phosphate dehydrogenase	8442.76	1.39	CM
**P0C0S7**	Histone H2A.Z	836.06	1.26	CM
**P47858**	ATP-dependent 6-phosphofructokinase_ muscle type	48.95	1.54	CM
**Q5MYU0**	2'-5'-oligoadenylate synthase 2	48.89	1.34	CM
**P54311**	Guanine nucleotide-binding protein G(I)/G(S)/G(T) subunit beta-1	222.87	1.48	CM
**P54313**	Guanine nucleotide-binding protein G(I)/G(S)/G(T) subunit beta-2	218.32	1.51	CM
**Q00715**	Histone H2B type 1	1856.85	1.45	CM
**O35353**	Guanine nucleotide-binding protein subunit beta-4	185.04	1.35	CM
**P62804**	Histone H4	1422.57	1.26	CM
**P59215**	Guanine nucleotide-binding protein G(o) subunit alpha	1196.22	1.52	CM
**Q9ESV6**	Glyceraldehyde-3-phosphate dehydrogenase_ testis-specific	1155.82	1.46	CM
**P35289**	Ras-related protein Rab-15	1036.14	1.36	CM
**P0C169**	Histone H2A type 1-C	1024.05	1.27	CM

**Q64598**	Histone H2A type 1-F	1024.05	1.27	CM
**P0CC09**	Histone H2A type 2-A	1024.05	1.27	CM
**Q00728**	Histone H2A type 4	1024.05	1.27	CM
**P02262**	Histone H2A type 1	1024.05	1.26	CM
**P0C170**	Histone H2A type 1-E	1024.05	1.26	CM
**Q4FZT6**	Histone H2A type 3	1024.05	1.26	CM
**A9UMV8**	Histone H2A.J	1024.05	1.26	CM
**Q6Q7Y5**	Guanine nucleotide-binding protein subunit alpha-13	1020.03	1.52	CM
**Q63210**	Guanine nucleotide-binding protein subunit alpha-12	1020.03	1.51	CM
**P06685**	Sodium/potassium-transporting ATPase subunit alpha-1	478.87	1.40	CM
**P06686**	Sodium/potassium-transporting ATPase subunit alpha-2	575.36	1.38	CM
**P06687**	Sodium/potassium-transporting ATPase subunit alpha-3	695.28	1.40	CM
**Q64541**	Sodium/potassium-transporting ATPase subunit alpha-4	254.19	1.35	CM
**P07340**	Sodium/potassium-transporting ATPase subunit beta-1	456.82	1.45	CM
**P16617**	Phosphoglycerate kinase 1	383.39	1.38	CM
**P09626**	Potassium-transporting ATPase alpha chain 1	50.98	1.39	CM
**P54708**	Potassium-transporting ATPase alpha chain 2	254.19	1.34	CM
**P84092**	AP-2 complex subunit mu	68.35	1.28	CM
**Q05982**	Nucleoside diphosphate kinase A	287.99	1.46	CM
**P19804**	Nucleoside diphosphate kinase B	287.99	1.48	CM
**P10111**	Peptidyl-prolyl cis-trans isomerase A	529.83	1.54	CM
**P25113**	Phosphoglycerate mutase 1	248.18	1.65	CM
**P48500**	Triosephosphate isomerase	1730.45	1.46	CM
**Q9QUL6**	Vesicle-fusing ATPase	51.69	1.49	CM
**P62815**	V-type proton ATPase subunit B_ brain isoform	185.22	1.51	CM
**Q1WIM3**	Cell adhesion molecule 3	41.12	1.28	CM
**P45592**	Cofilin-1	1580.13	1.49	CM
**P06761**	Endoplasmic reticulum chaperone BiP	234.63	1.55	CM
**P01946**	Hemoglobin subunit alpha-1/2	17560.29	1.12	CM
**P02091**	Hemoglobin subunit beta-1	6789.73	1.16	CM
**P62963**	Profilin-1	338.86	1.77	CR
**Q62952**	Dihydropyrimidinase-related protein 3	37.59	1.58	CR
**P05065**	Fructose-bisphosphate aldolase A	863.61	1.57	CR
**Q5XIF6**	Tubulin alpha-4A chain	9823.46	1.40	CR

**Q6P9T8**	Tubulin beta-4B chain	9552.34	1.42	CR
**P19332**	Microtubule-associated protein tau	90.16	1.26	CR
**Q68FR8**	Tubulin alpha-3 chain	8962.88	1.43	CR
**Q4QRB4**	Tubulin beta-3 chain	8913.07	1.43	CR
**P60711**	Actin_ cytoplasmic 1	7708.2	1.51	CR
**P63259**	Actin_ cytoplasmic 2	7698.71	1.52	CR
**Q6AY56**	Tubulin alpha-8 chain	6725.47	1.42	CR
**P15146**	Microtubule-associated protein 2	45.31	1.32	CR
**Q6P9V9**	Tubulin alpha-1B chain	12809.87	1.43	CR
**P68370**	Tubulin alpha-1A chain	12599.98	1.45	CR
**P85108**	Tubulin beta-2A chain	12233.2	1.43	CR
**Q3KRE8**	Tubulin beta-2B chain	12226.68	1.43	CR
**P11442**	Clathrin heavy chain 1	115.73	1.55	CR
**P69897**	Tubulin beta-5 chain	10835.71	1.42	CR
**Q6AYZ1**	Tubulin alpha-1C chain	10386.45	1.42	CR
**P09117**	Fructose-bisphosphate aldolase C	690.81	1.51	CR
**Q62950**	Dihydropyrimidinase-related protein 1	209.35	1.54	CR
**P47942**	Dihydropyrimidinase-related protein 2	1694.23	1.49	CR
**P21575**	Dynamin-1	128.86	1.42	CR
**P39052**	Dynamin-2	34.72	1.45	CR
**Q08877**	Dynamin-3	18.87	1.45	CR
**Q5RJQ4**	NAD-dependent protein deacetylase sirtuin-2	332.42	1.46	CR
**Q5RKI0**	WD repeat-containing protein 1	69.93	1.43	CR
**P62630**	Elongation factor 1-alpha 1	153.66	1.39	CR
**P62632**	Elongation factor 1-alpha 2	82.84	1.43	CR
**P15999**	ATP synthase subunit alpha_ mitochondrial	872.7	1.48	MA
**O88767**	Protein/nucleic acid deglycase DJ-1	746.41	1.22	MA
**P00507**	Aspartate aminotransferase_ mitochondrial	505.71	1.49	MA
**P08461**	Dihydrolipoyllysine-residue acetyltransferase component of pyruvate dehydrogenase complex_ mitochondrial	50.58	1.48	MA
**P07335**	Creatine kinase B-type	1516.55	1.40	MA
**Q05962**	ADP/ATP translocase 1	652.7	1.40	MA
**Q09073**	ADP/ATP translocase 2	565.81	1.46	MA
**P84079**	ADP-ribosylation factor 1	65.42	1.39	MA

**P84082**	ADP-ribosylation factor 2	65.42	1.32	MA
**P61206**	ADP-ribosylation factor 3	65.42	1.40	MA
**P61751**	ADP-ribosylation factor 4	47.49	1.26	MA
**P84083**	ADP-ribosylation factor 5	47.49	1.25	MA
**P10719**	ATP synthase subunit beta_ mitochondrial	2896.76	1.48	MA
**P31399**	ATP synthase subunit d_ mitochondrial	156.04	1.55	MA
**Q06647**	ATP synthase subunit O_ mitochondrial	278.89	1.51	MA
**Q9Z2L0**	Voltage-dependent anion-selective channel protein 1	287.96	1.46	MA
**P81155**	Voltage-dependent anion-selective channel protein 2	228.35	1.39	MA
**Q9R1Z0**	Voltage-dependent anion-selective channel protein 3	150.42	1.42	MA
**P16036**	Phosphate carrier protein_ mitochondrial	179.15	1.28	MA
**P26284**	Pyruvate dehydrogenase E1 component subunit alpha_ somatic form_ mitochondrial	38.87	1.62	MA
**P49432**	Pyruvate dehydrogenase E1 component subunit beta_ mitochondrial	246.76	1.57	MA
**P12928**	Pyruvate kinase PKLR	268.94	1.45	MA
**P11980**	Pyruvate kinase PKM	1534.5	1.40	MA
**Q9ER34**	Aconitate hydratase_ mitochondrial	187.21	1.54	MA
**P05708**	Hexokinase-1	32.41	1.43	MA
**P04642**	L-lactate dehydrogenase A chain	174.5	1.48	MA
**P42123**	L-lactate dehydrogenase B chain	196.55	1.35	MA
**P31000**	Vimentin	131.49	1.14	MA
**P25809**	Creatine kinase U-type_ mitochondrial	161.57	1.39	MA
**P37377**	Alpha-synuclein	271.99	1.68	NSR
**P13596**	Neural cell adhesion molecule 1	38.51	1.65	NSR
**Q63754**	Beta-synuclein	851.66	1.63	NSR
**P11730**	Calcium/calmodulin-dependent protein kinase type II subunit gamma	136.53	1.63	NSR
**P11275**	Calcium/calmodulin-dependent protein kinase type II subunit alpha	1107.35	1.62	NSR
**Q05175**	Brain acid soluble protein 1	94.21	1.62	NSR
**P08413**	Calcium/calmodulin-dependent protein kinase type II subunit beta	214.74	1.58	NSR
**Q63942**	GTP-binding protein Rab-3D	75.13	1.45	NSR

**P10860**	Glutamate dehydrogenase 1_ mitochondrial	56.67	1.49	NSR
**P68035**	Actin_ alpha cardiac muscle 1	3064.16	1.51	NSR
**P68136**	Actin_ alpha skeletal muscle	3059.82	1.51	NSR
**P62738**	Actin_ aortic smooth muscle	3035.28	1.54	NSR
**P63269**	Actin_ gamma-enteric smooth muscle	3035.28	1.52	NSR
**P15791**	Calcium/calmodulin-dependent protein kinase type II subunit delta	188.01	1.55	NSR
**P35284**	Ras-related protein Rab-12	1004.3	1.38	NSR
**P35280**	Ras-related protein Rab-8A	992	1.40	NSR
**P35281**	Ras-related protein Rab-10	992	1.39	NSR
**Q53B90**	Ras-related protein Rab-43	992	1.39	NSR
**Q6NYB7**	Ras-related protein Rab-1A	992	1.38	NSR
**P10536**	Ras-related protein Rab-1B	992	1.38	NSR
**P51156**	Ras-related protein Rab-26	992	1.38	NSR
**Q5U316**	Ras-related protein Rab-35	992	1.38	NSR
**P05714**	Ras-related protein Rab-4A	992	1.38	NSR
**P51146**	Ras-related protein Rab-4B	992	1.38	NSR
**P70550**	Ras-related protein Rab-8B	992	1.38	NSR
**P61107**	Ras-related protein Rab-14	992	1.36	NSR
**P02688**	Myelin basic protein	4721.22	-0.97	NSR
**P02688**	Myelin basic protein	4721.22	0.97	NSR
**P60203**	Myelin proteolipid protein	5849.56	1.20	NSR
**P07722**	Myelin-associated glycoprotein	44.41	1.32	NSR
**P16884**	Neurofilament heavy polypeptide	131.49	1.12	NSR
**P19527**	Neurofilament light polypeptide	139.69	1.26	NSR
**P12839**	Neurofilament medium polypeptide	131.49	1.20	NSR
**Q812E9**	Neuronal membrane glycoprotein M6-a	70.65	1.39	NSR
**P35286**	Ras-related protein Rab-13	137.44	1.48	NSR
**P63012**	Ras-related protein Rab-3A	228.54	1.42	NSR
**Q63941**	Ras-related protein Rab-3B	55.63	1.45	NSR
**P62824**	Ras-related protein Rab-3C	55.63	1.46	NSR
**Q9WVB1**	Ras-related protein Rab-6A	203.47	1.39	NSR
**P50398**	Rab GDP dissociation inhibitor alpha	191.83	1.51	NSR
**P50399**	Rab GDP dissociation inhibitor beta	133.66	1.54	NSR

**Q5XI73**	Rho GDP-dissociation inhibitor 1	322.17	1.52	NSR
**P63329**	Serine/threonine-protein phosphatase 2B catalytic subunit alpha isoform	54.99	1.57	NSR
**P20651**	Serine/threonine-protein phosphatase 2B catalytic subunit beta isoform	22.44	1.54	NSR
**P31044**	Phosphatidylethanolamine-binding protein 1	387.68	1.43	NSR
**Q62658**	Peptidyl-prolyl cis-trans isomerase FKBP1A	343.04	1.99	NSR
**P13233**	2'_3'-cyclic-nucleotide 3'-phosphodiesterase	638.75	1.22	NSR
**P11030**	Acyl-CoA-binding protein	882.98	1.57	NSR
**P04764**	Alpha-enolase	666.73	1.42	NSR
**P23565**	Alpha-internexin	212.03	1.34	NSR
**P13221**	Aspartate aminotransferase_ cytoplasmic	283.02	1.45	NSR
**O35095**	Neurochondrin	58.5	1.49	NSR
**P01830**	Thy-1 membrane glycoprotein	216.68	1.19	NSR
**Q00981**	Ubiquitin carboxyl-terminal hydrolase isozyme L1	551.92	1.48	NSR
**P15429**	Beta-enolase	209.91	1.38	NSR
**Q63198**	Contactin-1	48.87	1.27	NSR
**O35179**	Endophilin-A1	50.93	1.52	NSR
**P24942**	Excitatory amino acid transporter 1	132.53	1.55	NSR
**P31596**	Excitatory amino acid transporter 2	82.67	1.54	NSR
**P07323**	Gamma-enolase	719.63	1.49	NSR
**P47819**	Glial fibrillary acidic protein	180.63	1.27	NSR
**Q6P6V0**	Glucose-6-phosphate isomerase	131.54	1.49	NSR
**P08010**	Glutathione S-transferase Mu 2	24.2	1.84	RR
**P63039**	60 kDa heat shock protein_ mitochondrial	84.73	1.58	RR
**P63018**	Heat shock cognate 71 kDa protein	618.98	1.51	RR
**P26772**	10 kDa heat shock protein_ mitochondrial	488.77	1.48	RR
**P04905**	Glutathione S-transferase Mu 1	39.83	1.55	RR
**P55063**	Heat shock 70 kDa protein 1-like	35.76	1.55	RR
**P0DMW0**	Heat shock 70 kDa protein 1A	32.66	1.54	RR
**P0DMW1**	Heat shock 70 kDa protein 1B	32.66	1.52	RR
**P82995**	Heat shock protein HSP 90-alpha	239.47	1.35	RR
**P34058**	Heat shock protein HSP 90-beta	213.82	1.35	RR
**Q5XHZ0**	Heat shock protein 75 kDa_ mitochondrial	153.25	1.45	RR

**P14659**	Heat shock-related 70 kDa protein 2	147.3	1.49	RR
**P09606**	Glutamine synthetase	124.52	1.25	RR
**P04906**	Glutathione S-transferase P	118.27	1.23	RR
**P08009**	Glutathione S-transferase Yb-3	116.01	1.51	RR
**P07632**	Superoxide dismutase [Cu-Zn]	58.53	1.49	RR
**Q68FY0**	Cytochrome b-c1 complex subunit 1_ mitochondrial	143.73	1.46	RR
**P00406**	Cytochrome c oxidase subunit 2	105.65	1.23	RR
**P10888**	Cytochrome c oxidase subunit 4 isoform 1_ mitochondrial	99.64	1.48	RR
**P11240**	Cytochrome c oxidase subunit 5A_ mitochondrial	876.52	1.54	RR
**P12075**	Cytochrome c oxidase subunit 5B_ mitochondrial	292.65	1.51	RR
**P62898**	Cytochrome c_ somatic	313.89	1.46	RR
**Q63716**	Peroxiredoxin-1	101.4	1.63	RR
**P35704**	Peroxiredoxin-2	183.67	1.30	RR
**Q9R063**	Peroxiredoxin-5_ mitochondrial	559.67	1.32	RR
**O35244**	Peroxiredoxin-6	208.92	1.58	RR
**O88989**	Malate dehydrogenase_ cytoplasmic	595.14	1.46	RR
**P04636**	Malate dehydrogenase_ mitochondrial	1333.71	1.46	RR
**P09812**	Glycogen phosphorylase_ muscle form	16.73	1.63	RR
**P29101**	Synaptotagmin-2	35.7	1.79	SS
**P61765**	Syntaxin-binding protein 1	713.98	1.38	SS
**P11505**	Plasma membrane calcium-transporting ATPase 1	31.24	1.39	SS
**Q63537**	Synapsin-2	230.01	1.55	SS
**P07825**	Synaptophysin	192.41	1.48	SS
**P21707**	Synaptotagmin-1	183.1	1.43	SS
**P63102**	14-3-3 protein zeta/delta	1740.65	1.48	SS
**P09951**	Synapsin-1	160.54	1.49	SS
**P62260**	14-3-3 protein epsilon	1205.61	1.54	SS
**P68511**	14-3-3 protein eta	1131.05	1.46	SS
**P61983**	14-3-3 protein gamma	1131.05	1.46	SS
**P68255**	14-3-3 protein theta	1131.05	1.46	SS
**P35213**	14-3-3 protein beta/alpha	1131.05	1.43	SS
**P61265**	Syntaxin-1B	44.43	1.51	SS

[a] Accession ID According To Uniport.Org Database. Positive and negative values of fold change indicate up- and down-regulated proteins, respectively. Results of the comparison between the al group versus the control group. PLGS - Protein Lynx Global Server. FOLD CHANGE - measure describing the degree of quantity change

between final and original value. Legend: Cytoskeleton Regulation (CR); Cell Metabolism (CM); Mitochondrial Activity (MA); Redox Regulation (RR); Nervous System Regulation (NSR); Synaptic Signaling (SS).

Table S2. Identified proteins with exclusive regulation or absent in the hippocampus of rats exposed to aluminum.

Access Number [a]	Protein Name	PLGS Score	Fold Change	Biological Process
**Q9Z0U5**	Aldehyde oxidase 1	37.37	-	CC
**Q9WTP0**	Band 4.1-like protein 1	29.34	-	MF
**B2RYG7**	Docking protein 3	123.11	-	CC
**Q5XI74**	Endonuclease/exonuclease/phosphatase family domain-containing protein 1	39.12	-	CC, MF, BP
**Q62825**	Exocyst complex component 3	42.38	-	BP
**Q8K430**	Kelch-like protein 17	63.65	-	MF, BP
**Q63560**	Microtubule-associated protein 6	30.56	-	CC, MF, BP
**P21263**	Nestin	27.37	-	CC, MF, BP
**Q66HD3**	Nuclear autoantigenic sperm protein	25.62	-	CC, MF, BP
**O35264**	Platelet-activating factor acetylhydrolase IB subunit beta	166.46	-	CC, MF, BP
**P0CG51**	Polyubiquitin-B	368.81	-	CC, MF, BP
**Q63429**	Polyubiquitin-C	368.81	-	CC, MF, BP
**Q5XIH7**	Prohibitin-2	184.24	-	CC, MF, BP
**Q5BK81**	Prostaglandin reductase 2	68.36	-	MF, BP
**P04631**	Protein S100-B	473.48	-	CC, MF, BP
**Q5BJL5**	Protein strawberry notch homolog 1	23.29	-	CC, MF, BP
**O88664**	Serine/threonine-protein kinase TAO1	40.09	-	MF, BP
**Q3I408**	Solute carrier family 22 member 5	192.97	-	CC
**Q6DG50**	TANK-binding kinase 1-binding protein 1	34.36	-	BP
**P62982**	Ubiquitin-40S ribosomal protein S27a	368.81	-	MF
**P62986**	Ubiquitin-60S ribosomal protein L40	368.81	-	MF
**Q71LX6**	Xin actin-binding repeat-containing protein 2	25.07	-	MF
**Q5XI78**	2-oxoglutarate dehydrogenase_ mitochondrial	33.14	+	MF, BP
**Q5FVR4**	3'-5' exoribonuclease 1	62.59	+	MF, BP
**Q4V7C7**	Actin-related protein 3	107.54	+	MF, BP
**Q9Z286**	Adenylate cyclase type 10	63.37	+	MF, BP
**P51635**	Aldo-keto reductase family 1 member A1	106.48	+	MF, BP

**Q63028**	Alpha-adducin	98.31	+	CC
**P48037**	Annexin A6	47.85	+	CC, MF, BP
**Q05764**	Beta-adducin	74.65	+	MF
**P85969**	Beta-soluble NSF attachment protein	89.86	+	BP
**Q9JHX4**	Caspase-8	51.32	+	MF, BP
**Q62745**	CD81 antigen	54.82	+	CC
**Q8K3U6**	Coagulation factor VII	152.04	+	MF, BP
**Q66H73**	Coiled-coil domain-containing protein 82	70.02	+	CC
**Q0V8T4**	Contactin-associated protein like 5-3	43.15	+	BP
**Q8K4F8**	Cyclin-F	59.21	+	MF, BP
**P20788**	Cytochrome b-c1 complex subunit Rieske_ mitochondrial	203.74	+	MF, BP
**P11950**	Cytochrome c oxidase subunit 6C-1	155.75	+	CC
**P11951**	Cytochrome c oxidase subunit 6C-2	529.93	+	CC
**Q64559**	Cytosolic acyl coenzyme A thioester hydrolase	138.32	+	MF, BP
**B1H299**	DDB1- and CUL4-associated factor 17	87.27	+	BP
**Q8CF97**	Deubiquitinating protein VCIP135	46	+	MF, BP
**Q6TUG0**	DnaJ homolog subfamily B member 11	79.78	+	MF
**Q68SB1**	Double-stranded RNA-binding protein Staufen homolog 2	59.65	+	MF, BP
**P63170**	Dynein light chain 1_ cytoplasmic	509	+	MF, BP
**Q78P75**	Dynein light chain 2_ cytoplasmic	405.05	+	MF, BP
**O35964**	Endophilin-A2	72.86	+	BP
**O70513**	Galectin-3-binding protein	41.32	+	BP
**P08050**	Gap junction alpha-1 protein	162.29	+	CC
**P13264**	Glutaminase kidney isoform_ mitochondrial	89.5	+	MF
**O35077**	Glycerol-3-phosphate dehydrogenase [NAD(+)]_ cytoplasmic	106.27	+	MF
**Q9EQH1**	GRB2-associated-binding protein 2	57.32	+	CC, MF, BP
**P52287**	Guanine nucleotide-binding protein G(I)/G(S)/G(T) subunit beta-3	131.54	+	MF
**P51840**	Guanylyl cyclase GC-E	53.59	+	MF, BP
**Q71RP1**	Heparanase	99.81	+	MF, BP
**P27881**	Hexokinase-2	44.34	+	MF, BP
**Q9QYW1**	Intermediate conductance calcium-activated potassium channel protein 4	93.9	+	MF, BP
**Q99NA5**	Isocitrate dehydrogenase [NAD] subunit alpha_ mitochondrial	174.11	+	MF, BP

**Q68FX0**	Isocitrate dehydrogenase [NAD] subunit beta_ mitochondrial	101.15	+	BP
**Q6P6Q2**	Keratin_ type II cytoskeletal 5	169.1	+	CC, MF, BP
**Q2PQA9**	Kinesin-1 heavy chain	60.75	+	MF
**P70587**	Leucine-rich repeat-containing protein 7	43.66	+	CC, MF, BP
**P19629**	L-lactate dehydrogenase C chain	46.73	+	MF
**Q64122**	Myosin regulatory light polypeptide 9	172.33	+	MF
**P19234**	NADH dehydrogenase [ubiquinone] flavoprotein 2_ mitochondrial	166.54	+	MF, BP
**P07936**	Neuromodulin	126.05	+	MF, BP
**P84076**	Neuron-specific calcium-binding protein hippocalcin	88.29	+	CC, MF, BP
**F1M707**	Neutrophil cytosolic factor 1	54.59	+	CC
**Q811R2**	Peroxisome proliferator-activated receptor gamma coactivator 1-beta	47.89	+	CC
**Q6AYQ3**	Phenylalanine--tRNA ligase_ mitochondrial	62.69	+	MF, BP
**P16290**	Phosphoglycerate mutase 2	114.53	+	MF, BP
**B4F785**	Pikachurin	51.11	+	CC
**D4ACE5**	Protein inturned	177.77	+	MF, BP
**Q8VBU2**	Protein NDRG2	117.17	+	MF, BP
**P0C5X8**	Protein tweety homolog 1	94.22	+	MF, BP
**O35331**	Pyridoxal kinase	76.2	+	MF
**O35550**	Rab GTPase-binding effector protein 1	45.2	+	CC, MF, BP
**Q62956**	Receptor tyrosine-protein kinase erbB-4	91.94	+	MF, BP
**Q9JLT7**	Retinal homeobox protein Rx	77.9	+	MF, BP
**Q63965**	Sideroflexin-1	58.58	+	BP
**Q9JHY2**	Sideroflexin-3	76.45	+	BP
**D3ZQL7**	Similar to 25 kDa brain-specific protein (P25-alpha) (Predicted)_ isoform CRA_a	278.74	+	MF, BP
**P31647**	Sodium- and chloride-dependent GABA transporter 3	81.74	+	BP
**Q80ZA5**	Sodium-driven chloride bicarbonate exchanger	47.64	+	BP
**Q9QWN8**	Spectrin beta chain_ non-erythrocytic 2	43.43	+	MF
**P47861**	Synaptotagmin-5	37.67	+	CC, MF, BP
**Q5XIM9**	T-complex protein 1 subunit beta	38.27	+	MF
**Q10836**	Thyrotropin-releasing hormone-degrading ectoenzyme	45.36	+	MF
**P70566**	Tropomodulin-2	72.67	+	MF

**A0A0G2K2D6**	Tubulin polymerization-promoting protein	278.74	+	MF, BP
**Q5RJR2**	Twinfilin-1	69.44	+	MF
**Q9Z1A6**	Vigilin	55.82	+	MF

[a] Accession ID according to Uniport.org database. Sign of – and + indicate exclusive expression in the control (absent in exposed) or exposed group, respectively. Results of the comparison between the Al group versus the control group. PLGS - Protein Lynx Global Server. FOLD CHANGE - Measure describing the degree of quantity change between final and original value. Legend: Cellular Component (CC), Biological Process (BP) and Molecular Function (MF).

Table S3. List of proteins that interacted in the overrepresentation analysis (ORA) in the hippocampus of rats of $AlCl_3$ vs. Control group, based on biological processes of gene ontology.

Accession ID[a]	Protein Description
**O35244**	Peroxiredoxin-6
**O88767**	Parkinson disease protein 7 homolog
**O88989**	Malate dehydrogenase, cytoplasmic
**P00507**	Aspartate aminotransferase, mitochondrial
**P04636**	Malate dehydrogenase, mitochondrial
**P04642**	L-lactate dehydrogenase A chain
**P04764**	Alpha-enolase
**P06761**	Endoplasmic reticulum chaperone BiP
**P07335**	Creatine kinase B-type
**P07632**	Superoxide dismutase [Cu-Zn]
**P08461**	Dihydrolipoyllysine-residue acetyltransferase component of pyruvate dehydrogenase complex, mitochondrial
**P09117**	Fructose-bisphosphate aldolase C
**P10719**	ATP synthase subunit beta, mitochondrial
**P10888**	Cytochrome c oxidase subunit 4 isoform 1, mitochondrial
**P11240**	Cytochrome c oxidase subunit 5A, mitochondrial
**P11442**	Clathrin heavy chain 1
**P15429**	Beta-enolase
**P15999**	ATP synthase subunit alpha, mitochondrial
**P19234**	NADH dehydrogenase [ubiquinone] flavoprotein 2, mitochondrial
**P19804**	Nucleoside diphosphate kinase B
**P20788**	Cytochrome b-c1 complex subunit Rieske, mitochondrial

**P34058**	Heat shock protein HSP 90-beta
**P35213**	14-3-3 protein beta/alpha
**P35280**	Ras-related protein Rab-8A
**P35704**	Peroxiredoxin-2
**P42123**	L-lactate dehydrogenase B chain
**P48500**	Triosephosphate isomerase
**P49432**	Pyruvate dehydrogenase E1 component subunit beta, mitochondrial
**P59215**	Guanine nucleotide-binding protein G(o) subunit alpha
**P60711**	Actin, cytoplasmic 1
**P61765**	Syntaxin-binding protein 1
**P62260**	14-3-3 protein epsilon
**P62630**	Elongation factor 1-alpha 1
**P63039**	60 kDa heat shock protein, mitochondrial
**P63102**	14-3-3 protein zeta/delta
**P68035**	Actin, alpha cardiac muscle 1
**P68136**	Actin, alpha skeletal muscle
**P70550**	Ras-related protein Rab-8B
**Q05982**	Nucleoside diphosphate kinase A
**Q06647**	ATP synthase subunit O, mitochondrial
**Q08877**	Dynamin-3
**Q09073**	ADP/ATP translocase 2
**Q5XIM9**	T-complex protein 1 subunit beta
**Q63716**	Peroxiredoxin-1
**Q68FX0**	Isocitrate dehydrogenase [NAD] subunit beta, mitochondrial
**Q68FY0**	Cytochrome b-c1 complex subunit 1, mitochondrial
**Q6P9T8**	Tubulin beta-4B chain
**Q99NA5**	Isocitrate dehydrogenase [NAD] subunit alpha, mitochondrial
**Q9ER34**	Aconitate hydratase, mitochondrial
**Q9Z2L0**	Voltage-dependent anion-selective channel protein 1

[a] Accession ID according to Uniport.org database.
